# HGMB1 and RAGE as Essential Components of Ti Osseointegration Process in Mice

**DOI:** 10.3389/fimmu.2019.00709

**Published:** 2019-04-05

**Authors:** Claudia Cristina Biguetti, Franco Cavalla, Elcia Varize Silveira, André Petenuci Tabanez, Carolina Favaro Francisconi, Rumio Taga, Ana Paula Campanelli, Ana Paula Favaro Trombone, Danieli C. Rodrigues, Gustavo Pompermaier Garlet

**Affiliations:** ^1^Department of Biological Sciences, Bauru School of Dentistry, University of São Paulo, São Paulo, Brazil; ^2^Department of Conservative Dentistry, School of Dentistry, University of Chile, Santiago, Chile; ^3^Department of Biological and Allied Health Sciences, Universidade Sagrado Coração, Bauru, Brazil; ^4^Department of Bioengineering, University of Texas at Dallas, Dallas, TX, United States

**Keywords:** DAMPs, pre-clinical studies, inflammation, HMGB1, bioengineering, osseointegration, implants, osteoimmunology

## Abstract

The release of the prototypic DAMP High Mobility Group Box 1 (HMGB1) into extracellular environment and its binding to the Receptor for Advanced Glycation End Products (RAGE) has been described to trigger sterile inflammation and regulate healing outcome. However, their role on host response to Ti-based biomaterials and in the subsequent osseointegration remains unexplored. In this study, HMGB1 and RAGE inhibition in the Ti-mediated osseointegration were investigated in C57Bl/6 mice. C57Bl/6 mice received a Ti-device implantation (Ti-screw in the edentulous alveolar crest and a Ti-disc in the subcutaneous tissue) and were evaluated by microscopic (microCT [bone] and histology [bone and subcutaneous]) and molecular methods (ELISA, PCR array) during 3, 7, 14, and 21 days. Mice were divided into 4 groups: Control (no treatment); GZA (IP injection of Glycyrrhizic Acid for HMGB1 inhibition, 4 mg/Kg/day); RAP (IP injection of RAGE Antagonistic Peptide, 4 mg/Kg/day), and vehicle controls (1.5% DMSO solution for GZA and 0.9% saline solution for RAP); treatments were given at all experimental time points, starting 1 day before surgeries. HMGB1 was detected in the Ti-implantation sites, adsorbed to the screws/discs. In Control and vehicle groups, osseointegration was characterized by a slight inflammatory response at early time points, followed by a gradual bone apposition and matrix maturation at late time points. The inhibition of HMGB1 or RAGE impaired the osseointegration, affecting the dynamics of mineralized and organic bone matrix, and resulting in a foreign body reaction, with persistence of macrophages, necrotic bone, and foreign body giant cells until later time points. While Control samples were characterized by a balance between M1 and M2-type response in bone and subcutaneous sites of implantation, and also MSC markers, the inhibition of HMGB1 or RAGE caused a higher expression M1 markers and pro-inflammatory cytokines, as well chemokines and receptors for macrophage migration until later time points. In conclusion, HMGB1 and RAGE have a marked role in the osseointegration, evidenced by their influence on host inflammatory immune response, which includes macrophages migration and M1/M2 response, MSC markers expression, which collectively modulate bone matrix deposition and osseointegration outcome.

## Introduction

Ti-based devices, such as dental implants, are classically used in dentistry, due to their osseointegration capacity that is translated into remarkable clinical success (1–3). However, understanding of the molecular interactions at Ti/host interface, which drive a beneficial equilibrium between immune/inflammatory response and the subsequent bone apposition toward Ti surface remains unclear (3).

A recent study performed an extensive molecular and histological characterization of Ti mediated osseointegration in C57Bl/6 mice, demonstrating a highly orchestrated and transient inflammatory response coordinated with the early stages of osseointegration (4). In view of the dominance of innate immunity elements in the host response that paves the way for osseointegration, in a process where numerous inflammation- and bone healing-related molecules are up-regulated (5, 6), macrophages have been regarded as central determinants of osseointegration outcome (7, 8). Indeed, macrophages can exert key regulatory functions by secreting a range of different mediators (chemokines, cytokines, enzymes, and growth factors) in the inflammatory microenvironment, which consequently influence the intensity and duration of immune response, affecting healing (9, 10). Recent studies suggest macrophages polarization into M1 or M2 phenotypes as a crucial step for determining the success or failure of biomaterial osseointegration, since the dominance of a M1-type response is related to chronic inflammation and fibrous encapsulation of Ti instead of successful osseointegration (7, 9, 11, 12).

Therefore, initial steps of the host inflammatory immune response that will shape macrophages fate in the biomaterial-implantation site seem an essential component for a successful osseointegration outcome. Macrophage polarization around biomaterials begins immediately post-implantation, with biomaterial surface recognition and a transient polarization state, which are influenced by varying microenvironmental cues, some of which are biomaterial-based (9). Thus, it has been supposed that the type and quantity of proteins adsorbed on a biomaterial is influenced by its surface morphological cues and chemistry, which may affect its recognition by macrophages consequently influencing their phenotypic polarization (9, 13).

Considering the candidate proteins for adsorption on Ti surface, damage associated molecular patterns (DAMPs) are a group of endogenous intracellular or extracellular molecules, which are released from their original sites into the microenvironment upon breakage of tissue components caused by trauma or stress, acting as local “danger signals” that trigger host response (14, 15). After their release from damaged tissues, DAMPs are recognized by a number of pattern recognition receptors (PRRs) primarily expressed on macrophages (10, 16, 17). Among several DAMPs/PRRs pathways already described in the literature, the interaction of High Mobility Group Box 1 (HMGB1), the prototypical and most well-characterized DAMP, with the Receptor for Advanced Glycation End Products (RAGE), has been associated with the activation of inflammatory responses and wound healing (18, 19). Indeed, while HMGB1, alone or associated with other molecules, can play pleiotropic functions by activating multiple receptors (TLR4 and TLR2, RAGE, CXCR4) (18, 20, 21). It is also important to mention that HMGB1 is a redox-sensitive molecule and consequently, redox status of its cysteine residues (Cys23, Cys45, and Cys106) is strongly affected by a pro-oxidative and pro-inflammatory environment, since various reactive oxygen species (ROS) are released in inflammatory environments (22, 23). Then, biphasic actions on HMGB1 (pro-inflammatory activity or immune tolerance/healing) may depends on the environment where this molecule is released. In this context, it has been suggested that oxidized or reduced forms of HMGB1 might differently affect the HMGB1 binding into different receptors and induce that biphasic actions (23). For example, oxidized form of HMGB1 accumulates during resolution of inflammation and tissue regeneration in liver, serving as a feedback mechanism to control its proinflammatory activity (22).

RAGE constitute the major receptor for HMGB1 (24–26). Importantly, the axis HMGB1/RAGE is related with several cellular effects which are important to inflammatory and healing outcomes, such as induction of inflammatory response and angiogenesis, tissue remodeling, and stimulation of cellular differentiation for regeneration (19, 27–31). In the context of M1/M2, evidence from *in vivo* studies point that HMGB1 can facilitate M1 macrophage phenotype in certain inflammatory disease models (32, 33), mainly based on HMGB1 interactions with TLR receptors (32). However, other *in vitro* (26) and *in vivo* disease models (34, 35) suggested that HMGB1 can enhance the activity of M2 macrophages, especially in a manner RAGE-dependent (26, 35). Importantly, despite the growing focus on macrophages role in healing, HMGB1/RAGE is a potential trigger of the overall host inflammatory immune response at biomaterials implantation sites, which theoretically can involve other cell besides the macrophages. Indeed, is still unclear how HMGB1/RAGE can trigger and regulate host responses in different inflammatory contexts.

Considering the influence of DAMPs regulating biomaterial incorporation, it has been demonstrated by *in vitro* studies that remaining HMGB1 within xenogeneic biologic scaffolds (after manufacturing processes) affects the response of monocytes/macrophages to the biomaterial and consequently can affect the inflammatory response, such as a bioactive molecule (36). On the other hand, in metallic and permanent biomaterial incorporation, the molecules driving the host response are theoretically exclusively released by host, such as hypothesized by recent reviews in biomaterials science literature (15, 37). Therefore, DAMPs are suggested to be released from tissue damage immediately after biomaterial implantation, possibly interacting with the surface and influencing the innate inflammatory response in the site of biomaterial implantation (15, 38). However, no previous studies have demonstrated the presence of endogenous DAMPs in biomaterials implantation sites, as well their putative role remains to be demonstrated in a cause-and-effect manner.

In face of all evidences for the role of HMGB1 and its cognate receptor RAGE in modulating inflammatory and healing responses, the release of HMGB1 after Ti implant placement could be a critical step for triggering inflammation and healing outcomes in osseointegration sites. Thus, in this present study, we investigated the role of HMGB1 during Ti-mediated oral osseointegration in C57Bl/6 mice, by means of a cause-effect study of pharmacological inhibition of HMGB1 or its cognate receptor RAGE.

## Materials and Methods

### Material Preparation

Titanium implant screws (titanium-6 aluminum-4 vanadium alloy, NTI-Kahla GmbH Rotary Dental Instruments, Kahla, Thüringen, Germany) of Ø 0.6 mm were cut at a length of 1.5 mm. Also, machined 6AL-4V Tinanium discs (Ti-discs) of Ø 6 and 2 mm thick from commercially pure grade 2 alloy were used for subcutaneous implantation. All material were sterilized by autoclaving before surgical procedures, as previously described for oral osseointegration model in C57Bl/6 mice (4).

### Animals

Experimental groups comprised C57Bl/6 male mice (10-weeks-old, 25 g of weight in average), bred and maintained in the animal facilities of University of São Paulo, cared according to the recommendations in the Guide for the Care and Use of Laboratory Animals of the National Institutes of Health (39) were used in this study. The experimental protocols were performed according to ARRIVE guidelines (40) and National Institutes of Health guide for the care and use of Laboratory animals (NIH Publications No. 8023, revised 1978), with approval by the local Institutional Committee for Animal Care and Use (CEEPA-FOB/USP, #012/2014). Mice were provided sterile water *ad libitum* and were fed with sterile standard solid mice chow (Nuvital, Curitiba, PR, Brazil) during all experimental periods of this study, except throughout the first 72 h post-Ti implantation for oral osseointegration model, in which diet was crumbled. Experimental groups for oral osseointegration were comprised by 10 animals per group/time point (3, 7, 14, and 21 days), with 6 animals per group/time point for microscopic analysis (microCT, histological, and birefringence analysis) and 4 for molecular (Real Time PCR array) assays; an additional 1 day time point group with 6 animals per group was used for protein elution and HMGB1 quantification. Experimental groups for subcutaneous Ti disc implantation were comprised by 5 animals per group/time point (3, 7, and 14 days) and Ti-disc was implanted in left and right side of animal dorsa, comprising 10 biological samples for each group/time point: 5 Ti disc samples (Ti discs containing the surrounding tissues) from the left side for microscopic analysis (histological, birefringence analysis, and immunohistochemistry) and 5 Ti disc samples from the right side for molecular analysis (Real Time PCR array) and protein elution (an additional 1 day time point was evaluated for HMGB1 quantification). All experimental groups (oral osseointegration and subcutaneous implantation) were divided according to each treatment: Control (no treatment); GZA, IP injection of glycyrrhizic acid (Sigma Aldrich) 200 mg/Kg/24 h for HMGB1 inhibition; vehicle control for GZA (intraperitoneal [IP] injection of 1.5% DMSO solution); RAP, IP injection of RAGE antagonistic peptide (RAP, Merck Millipore, USA) 4 mg/Kg/24 h as previously described (41, 42); and vehicle control for RAP, IP injection of saline solution 0.9%. Mice received daily IP injections of drugs/vehicle, starting 1 day before the surgical procedure and continuing toward the end of experimental periods. No antibiotics and anti-inflammatory drugs were administered to the animals after implantation surgery, in order to avoid interferences on investigated inflammatory/immunological pathways (4).

### Experimental Protocol for Oral Osseointegration Model

The Ti-implant placement in edentulous alveolar crest of the oral cavity of C57Bl/6 mice was performed as previously described (4, 43). Briefly, mice were anesthetized previous to the surgery by ketamine chloride 80 mg/kg (Dopalen, Agribrands Brasil, Paulínia, SP, Brazil) and xylazine chloride 160 mg/kg (Anasedan, Agribrands Brasil, Paulínia, SP, Brazil). Then, the mouse was placed in dorsal decubitus position under a stereomicroscope (DF Vasconcellos, São Paulo, SP, Brazil), and oral mucosa was cleaned using topical chlorhexidine solution for 1 min. An incision of 2 mm width parallel to the palatal crease and 1 mm in front of the left first maxillary molar was made and the subjacent bone was drilled using a Ø 0.50 mm pilot drill (NTI-Kahla GmbH Rotary Dental Instruments, Kahla, Thüringen, Germany) at 600 rpm using a surgical motor (NSK-Nakanishi International, Kanuma, Tochigi, Japan). The Ti-implant was screwed down in the implant bed using a castro viejo micro needle holder (Fine Science Tools, British Columbia, CA, USA). The right edentulous alveolar crest was used as Control side, without implant placement. Importantly, animals with early failure related to the surgical procedure (loss of primary stability upon placement) were immediately detected and were not included in the sample size; being only Ti implantations with complete absence of device mobility included in the sample for subsequent analysis, as previously characterized (4). At the end of experimental periods, mice were euthanized and maxillae were removed for microscopic (microtomographic, histological, histomorphometric) or molecular analysis. Samples selected for microscopic analysis were fixed in PBS-buffered formalin (10%) solution (pH 7.2) for 48 h at room temperature, washed overnight in running water and maintained in alcohol fixative (70% hydrous ethanol) until the conclusion of the μCT scanning. Then, the specimens were decalcified in 4.13% EDTA (pH 7.2) following histological processing protocols. Samples for molecular analysis were stored in RNA later (Ambion, Austin, TX, USA) solutions following previous protocols (44, 45), samples for HMGB1 quantification were submitted to protein elution protocol and subsequently frozen for posterior protein assay (46, 47).

### Experimental Protocol for Ti Implantation on Subcutaneous Tissue

Mice were anesthetized as previous described for oral osseointegration model. Then, a longitudinal incision was performed in the animal dorsa, were one Ti-disc was implanted in each side. Immediately down from Ti implantation, while the control region remained intact. Ti discs containing the surrounding tissues, as well control samples were collected from the left side for microscopic and from the right side for molecular analysis (Real Time PCR array). Samples collected for microscopic analysis were fixed in PBS-buffered formalin (10%) solution (pH 7.2) for 24 h at RT, then washed over-night in running water and processed for routine histology. Samples collected for molecular analysis were stored in RNAlater (Ambion, Austin, TX, USA) solutions for Real Time PCR array. For protein assay (i.e., HMGB1 detection), Ti-screws, and Ti-discs retrieved after implantation were submitted to protein elution protocol for posterior protein assay (46, 47).

### ELISA Assay for HMGB1 Detection

Ti-screws (implanted in bone) and Ti-discs (implanted into subcutaneous tissue) were retrieved from implantantion sites at different time points submitted to a protein elution protocol (46, 47). Briefly, Ti devices were subjected to five consecutive washes with 200 μl of double-distilled water and a final wash with 100 mM NaCl in 50 mM Tris-HCl to remove unadsorbed proteins. The absorbed proteins eluate was obtained by three consecutive submersions of the devices in a solution containing 4% SDS, 100 mM DTT, and 0.5 M TEAB, as previously (46, 47). Total protein of the serum was quantified for subsequent normalization (Pierce Protein Assay Kit), and HMGB1 was measured by ELISA according to the protocol recommended by the manufacturer (MyBioSource). The results were expressed as mean values ± standard deviation nanogram (ng) of protein per milligram of tissue, and represent values of duplicates of each sample obtained in two independent experiments.

### Micro-Computed Tomography (μCT) Assessment

Mice maxillae containing the Ti-implants were scanned by Skyscan 1176 System (Bruker Microct, Kontich, Belgium) at 80 kV, 300 μA, 180 degrees of rotation, and exposure range of 1 degree. After scanning and previous reconstructions (NRecon software, Bruker Microct, Kontich, Belgium), representative three-dimensional images were obtained by CT-Vox 2.3 software, while quantitative evaluation of bone to implant interface was assessed using CTAn 1.1.4.1 software (Bruker Microct, Kontich, Belgium) based in previous standardization for measuring bone implant contact volume by means of microCT (4). Briefly, for quantification of bone volume proportion (BV/TV, %) at the implant-bone interface area, a cylindrical region of interest (ROI) with a diameter of 700 μm was set and the bone volume quantification was performed only considering bone implant contact region. After binarization and separation between titanium body and bone by the difference of hyperdensities, BV/TV was acquired.

### Histomorphometry

The mice maxillae used for microCT scanning were processed for histological analysis following standardized procedures (4, 45, 48). For both, osseointegration model (maxillae) and subcutaneous, semi-serial sections considering the implantation area were cut with 4 μm thickness. A total of six samples (biological replicates) and nine semi-serial sections (technical replicates) from the central region of implantation sites in the maxilla were taken for hematoxylin and eosin [H&E] staining. For subcutaneous sites, a total of five samples (biological replicates) and eight semi-serial sections (technical replicates) were considered for histomorphometry. The histomorphometry was performed by a single calibrated investigator with a binocular microscope (Olympus Optical Co., Tokyo, Honshu, Japan) using a 100x immersion objective. Six histological fields *per* each HE section, comprising the region adjacent to thread spaces (for osseointegration) or Ti disc space (for subcutaneous), were observed under a 100 points grid in a quadrangular area, by using Image J software (Version 1.51, National Institutes of Health, Bethesda, MD, USA). Points were quantified coinciding with the following structures found in the osseointegration sites or in implant failure sites: blood clot, inflammatory cells, blood vessels, fibroblasts and fibers, osteoblasts, osteoclasts, bone matrix, necrotic bone and foreign body giant cells (FBGC), and other elements (empty spaces left by implant space). For subcutaneous, were quantified structures involving inflammatory and healing process surrounding the Ti-disc space (presence of blood clot, inflammatory cells, fibers, fibroblasts, and blood vessels). Results were presented as the mean area density for each structure considered in each examined group.

### Birefringence Analysis

A total of six different samples (biological replicates) and four semi-serial sections (technical replicates) for each sample were used for picrosirius red staining and birefringence analysis of the osseointegration model in the maxillae. For each semi-serial section, three histological fields were evaluated comprising the central region of bone to implant contact. In subcutaneous tissue, five samples (biological replicates) and four semi-serial sections (technical replicates) for each sample were analyzed. For each section, six histological fields were analyzed surrounding the Ti disc space. All specimens were analyzed at 40x magnification through polarizing lens coupled to a binocular inverted microscope (Leica DM IRB/E, Leica Microsystems Wetzlar GmbH, Wetzlar, Germany) and images were captured with a Leica Imaging Software (LAX, Leica Microsystems Wetzlar GmbH, Wetzlar, Germany). As previously described (4, 45, 48), green birefringence color indicates thin fibers; yellow and red colors at birefringence analysis indicate thick collagen fibers. Three fields from each section were analyzed through polarizing lens coupled to a binocular inverted microscope (Leica DM IRB/E, Leica Microsystems Wetzlar GmbH, Wetzlar, Germany), by using 40x magnification immersion objective. Images were captured with a Leica Imaging Software (LAX, Leica Microsystems Wetzlar GmbH, Wetzlar, Germany) and the quantification of birefringence brightness was performed using the software AxioVision 4.8 (Carl Zeiss Microscopy GmbH, Jena, Germany) considering green, yellow, and red spectra pixels2. Mean values of four sections from each animal were calculated and submitted to statistical analysis.

### Immunohistochemistry and Quantification of Immunolabeled Inflammatory Cells

A total of five samples (biological replicate) from subcutaneous tissue and three semi-serial sections (technical replicate) of each sample surrounding the Ti implant were used for individual immunodetection of Ly6g-GR1 (sc-168490), F4/80 (a pan marker for murine macrophages, sc-26642), CD80 (M1 macrophage, sc-376012), and CD206 (M2 macrophage, sc-34577), all primary antibodies purchased from Santa Cruz Biotechnology (Santa Cruz Biotechnology, Santa Cruz, CA, USA). Immunohistochemistry protocol was performed as previously described (48). Briefly, histological sections were rehydrated and retrieved the antigens by boiling the histological slides in 10 mM sodium citrate buffer pH 6 for 30 min at 100°C. Subsequently, the sections were pre-incubated with 3% Hydrogen Peroxidase Block (Spring Bioscience Corporation, CA, USA) and subsequently incubated with 7% NFDM to block serum proteins. All primary antibodies were diluted at 1:100 in diluent solution for 1 h at room temperature. Universal immuno-enzyme polymer method was used and sections were incubated in immunohistochemical staining reagent for 30 min at room temperature. The identification of antigen–antibody reaction was performed using 3-3'-diaminobenzidine (DAB) and counterstaining with Mayer's hematoxylin. Positive controls were performed by using mouse spleen for F4/80, CD80, and CD206 macrophages while Ly6g-Gr1+ were directly visualized in the inflamed tissues post-surgical trauma. The analysis of immunolabeled cells (Gr, F4/80, CD80, CD206) was performed by a single calibrated investigator using a 100x magnification, considering six histological fields per section, comprising subcutaneous tissue surrounding the Ti-disc. Three samples (biological replicate) for each experimental period and strains were used for quantitative analysis and a total of three sections of each biological replicate were quantified. A grid image was superimposed on the histological photomicrographs, with 10 parallel lines and 100 points in a quadrangular area, by using Image J software (Version 1.51, National Institutes of Health, Bethesda, MD, USA). Only the points coincident with the immunolabeled cells were considered in cell counting and the mean for each section was obtained for statistical analysis.

### Real Time PCR Array Reactions

Maxillae and subcutaneous tissue from all experimental groups and time points were dissected and samples containing only the region of the implant bed were storage in RNA stabilization solution (RNAlater, Thermofisher, Waltham, MA, USA) until Real Time PCR array reactions. Samples from the right side (without implant placement) of maxillae and samples from the down right side of subcutaneous tissue (control region remained intact) were used and a Control. Real Time PCR array reactions were performed as previously described (4, 44, 45), using initially a pool of four samples (biological replicates) from all experimental time-points for each group for maxilla and four samples (biological replicates) for subcutaneous tissue. For all experiments, were performed two technical replicates. Pool analysis were performed in order to select targets in which expression variation presented a significant variation compared to the Control side. Subsequently, upregulated targets were analyzed regarding their kinetics of expression for specific time points (3, 7, 14, and 21days) after implant placement. Briefly, the extraction of total RNA from implantation sites and controls was performed with RNeasy kit (Qiagen Inc, Valencia, CA, USA) according to manufacturers' instructions. The integrity of RNA samples was checked by 2100 Bioanalyzer (Agilent Technologies, Santa Clara, CA, USA), and the complementary DNA was synthesized using 3 μg of RNA through a reverse transcription reaction (QuantiTectRTkit, Qiagen Inc, Valencia, CA, USA) (44). The Real Time PCR array was performed in a Viia7 instrument (LifeTechnologies, Carlsbad, CA, USA) using custom panels for “wound healing” (PAMM-121), “inflammatory cytokines and receptors” (PAMM-011), and “Osteogenesis” (PAMM-026) (SABiosciences, Frederick, MD, USA) for gene expression profiling, followed by data analysis with the RT2 Profiler software (SABiosciences, Frederick, MD, USA) for normalizing the initial geometric mean of three constitutive genes (GAPDH, ACTB, Hprt1), following normalizing the Control group; as previously described (4). Data are expressed as heat map fold change relative to the Control group.

### Statistical Analysis

Statistical treatment of quantitative data was performed using GraphPad Prism 5.0 software (GraphPad Software Inc., San Diego, CA, USA). Normally distributed data were analyzed using ANOVA followed by Bonferroni's multiple comparison *post-hoc* tests or student's *t*-test where applicable. For non-normal distributions, data were analyzed by means Kruskal-Wallis test (followed by Dunn's test) and Mann-Whitney test. The statistical significance of the experiment involving Real Time PCR array was evaluated by the Mann-Whitney test, and the values tested for correction of Benjamini and Hochberg (49). Values of *p* < 0.05 were considered statistically significant.

## Results

### Detection of HMGB1 on Sites of Bone and Subcutaneous Implantation

**Figure 1:**
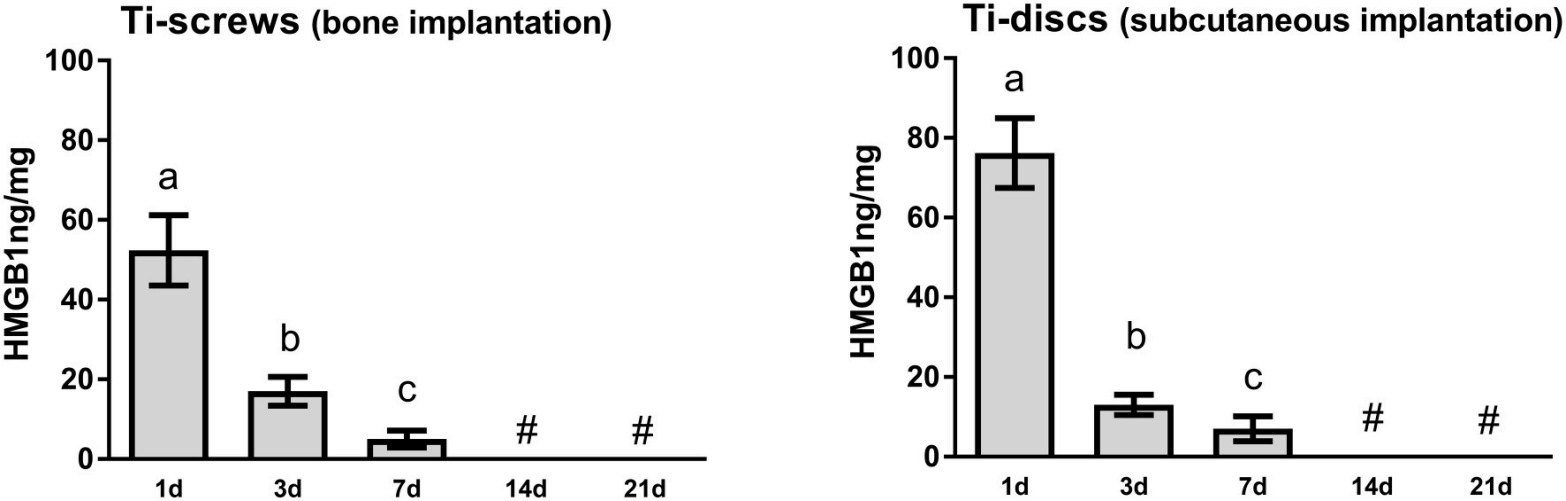
HMGB1 detection in the sites of bone Ti implantation. Ti-screws (implanted in bone) and Ti-discs (implanted into subcutaneous tissue) were retrieved from implantantion sites at 1, 3, 7, 14, and 21 days. Samples were submitted to a protein elution protocol followed by the HMGB1 quantification by ELISA according to the protocol recommended by the manufacturer (MyBioSource). The results were expressed as mean values ± standard deviation nanogram (ng) of protein per milligram of tissue, from a total of five animals/samples (biological replicates) and two technical replicates *per* each group and time point. Different letters indicate significant statistical differences (*p* < 0.05) among time periods in each group, symbol #represent “undectable levels” (Kruskal-Wallis followed by Dunn's test).

HMGB1 was found to be present in the protein adsorption layer characteristically formed in biomaterials surface after implantation (Figure 1), as demonstrated by the protein elution from both Ti-screws implanted in bone and Ti-discs implanted in subcutaneous tissue. HMGB1 was present in relatively high concentration in the 1 d time point, followed by a gradual decrease in 3 and 7 days' time points, being non-detectable at the 14 and 21 days' time-points (Figure 1), being this pattern similar in bone and subcutaneous implantation sites.

### μCT Assessment of Osseointegration

**Figure 2:**
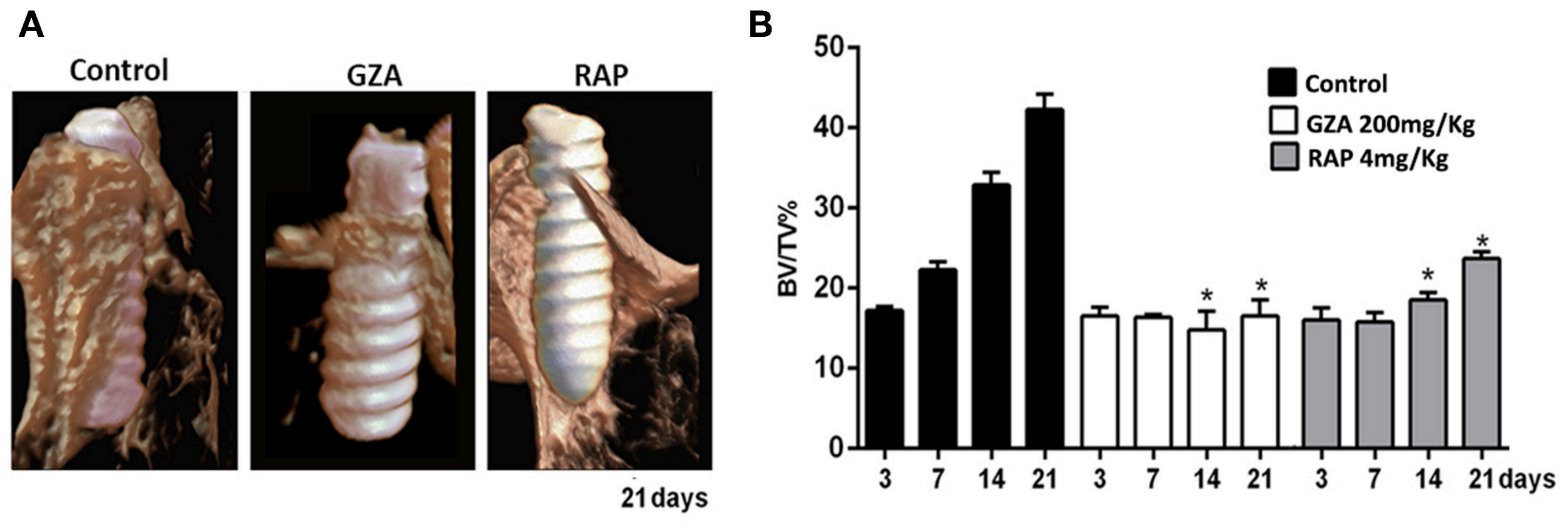
Micro-computed tomography (μCT) analysis of oral osseointegration model in C57Bl/6 mice under HMGB1 or RAGE inhibition. Mice received Ti-screw implantation in the edentulous ridge of maxilla and were divided in according to each treatment: Control (C group, with no treatment); Glycyrrhizic Acid at a dosage of 200 mg/Kg/day (GZA group); or RAGE antagonistic peptide at dosage of 4 mg/Kg/day (RAP group). **(A)** Three-dimensional representative images obtained with the CT-Vox software at 21 days post Ti implantation from Control, GZA, and RAP groups. **(B)** Quantitative analysis of bone volume/tissue volume (BV/TV, %) in the interface bone-Ti along days 3, 7, 14, and 21 post implantation for Control, GZA, and RAP groups. Results are presented as the mean and SD from a total of six biological replicates from each group and time point. Symbol ^*^indicates significant statistical differences (*p* < 0.05) in comparison with control.

Qualitative and quantitative analyses of mineralized bone matrix revealed a non-significant quantity of bone around Ti threads at 3 days among all groups, whose bone detected around Ti threads characterized the native bone supporting the Ti-implant (Figures 2A,B). Detectable, but not statistically significant newly formed bone matrix was observed at 7 days (22.33 ± 1.93) compared to 3 days (17.18 ± 1.11) post Ti-implantation in the Control group, and osseointegration was achieved throughout a gradual and proportion of bone apposition (BV/TV, %) around implant threads at 14 days (32.88 ± 3.16%) and 21 days (42.25 ± 3.86%; Figure 2B). On the other hand, the inhibition of HMGB1 and RAGE, in GZA and RAP treated animals, showed a significantly reduced BV/TV around Ti threads at 14 and 21 days compared to the Control group (Figure 2B), and DMSO and Saline Solution vehicles treated group as well (data not shown). The mean of BV/TV around implant threads in the GZA treated animals was 14.76 ± 4.06% at 14 days and 16.58 ± 3.40% at 21 days, while in RAP treated animals was 18.53 ± 1.60% at 14 days and 23.69 ± 1.40% at 21 days. The GZA and RAP vehicle control treated groups also achieved osseointegration with no statistical differences compared to the Control (data not shown).

### Birefringence of Collagen Fibers on Granulation Tissue and Bone Matrix During Osseointegration

**Figure 3:**
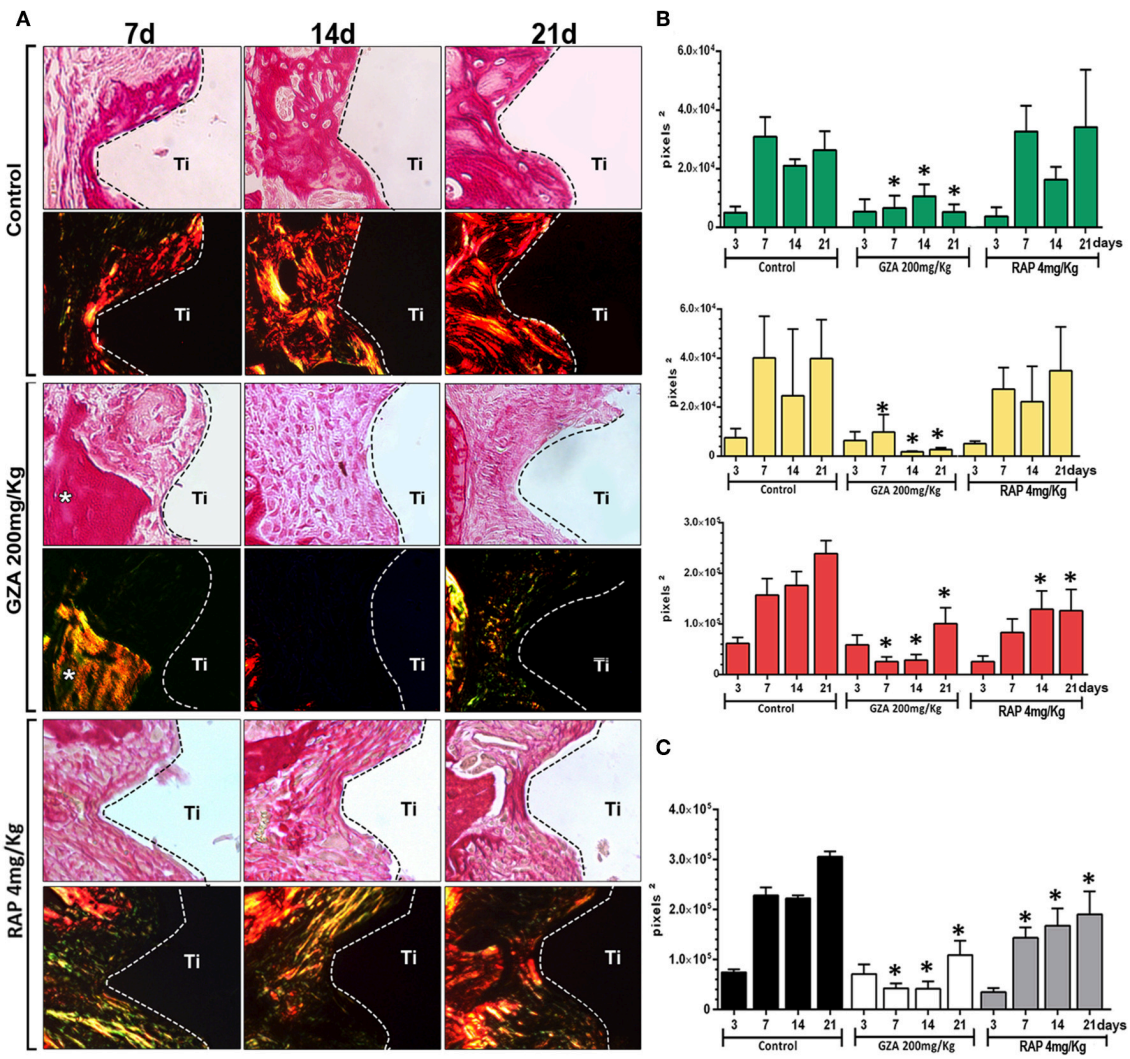
Birefringence analysis of collagen fibers along osseointegration model in C57Bl/6 mice under HMGB1 or RAGE inhibition. Mice received Ti-screw implantation in the edentulous ridge of maxilla and were divided in according to each treatment: Control (C group, with no treatment); Glycyrrhizic Acid at a dosage of 200 mg/Kg/day (GZA group); or RAGE antagonistic peptide at dosage of 4 mg/Kg/day (RAP group). **(A)** Representative sections from oral osseointegration process upon polarized and conventional light, to evaluate collagen fibers maturation along days 3, 7, 14, and 21 post-Ti-screw implantation in the different experimental groups. As visualized upon polarized light, green birefringence color indicates thin fibers; yellow and red colors at birefringence analysis indicate thick collagen fibers. Original magnification 40x. **(B)** Intensity of birefringence measured from Image-analysis software (AxioVision, v. 4.8, CarlZeiss) to identify and quantify area of collagen from each birefringence color (pixels 2) and **(C)** total area of collagen fibers (pixel2) throughout experimental periods. Results are presented as the mean and SD of pixels2 for each color in the birefringence analysis, from a total of six animals/samples (biological replicates) and four technical replicates *per* each group and time point. Symbol ^*^indicates a statistically significant difference vs. control (*p* < 0.05).

To comprehensively analyze the impact of HGMB1 or RAGE inhibition on organic bone matrix maturation on oral osseointegration in mice, we quantified green, yellow and red spectrum fibers from the bone matrix and initial granulation tissue for all groups (Figures 3A,B). All groups showed a negligible quantity of collagen fibers starting at 3 days around the Ti threads, emitting birefringence in the green spectrum (i.e., immature and thinner fibers). From 7 to 21 days, the Control group showed a significant increase in yellow and red collagen fibers, suggesting organic bone matrix maturation. Conversely, inhibition of HMGB1 in GZA treated mice caused a drastic impairment of bone collagen fibers formation, with significantly reduced amount of all birefringent type of fibers from 7 to 21 days compared to the Control. Under inhibition of RAGE (RAP treated mice), there was also impaired formation and maturation of collagen fibers, with a significantly reduced amount of total fibers at 14 and 21 days compared to the Control. No significant differences were observed in the dynamics of collagen fibers formation and maturation during osseointegration between GZA and RAP Control vehicle treated groups (data not shown).

### Histopathological Description and Histomorphometry of Healing Components During Osseointegration

**Figure 4:**
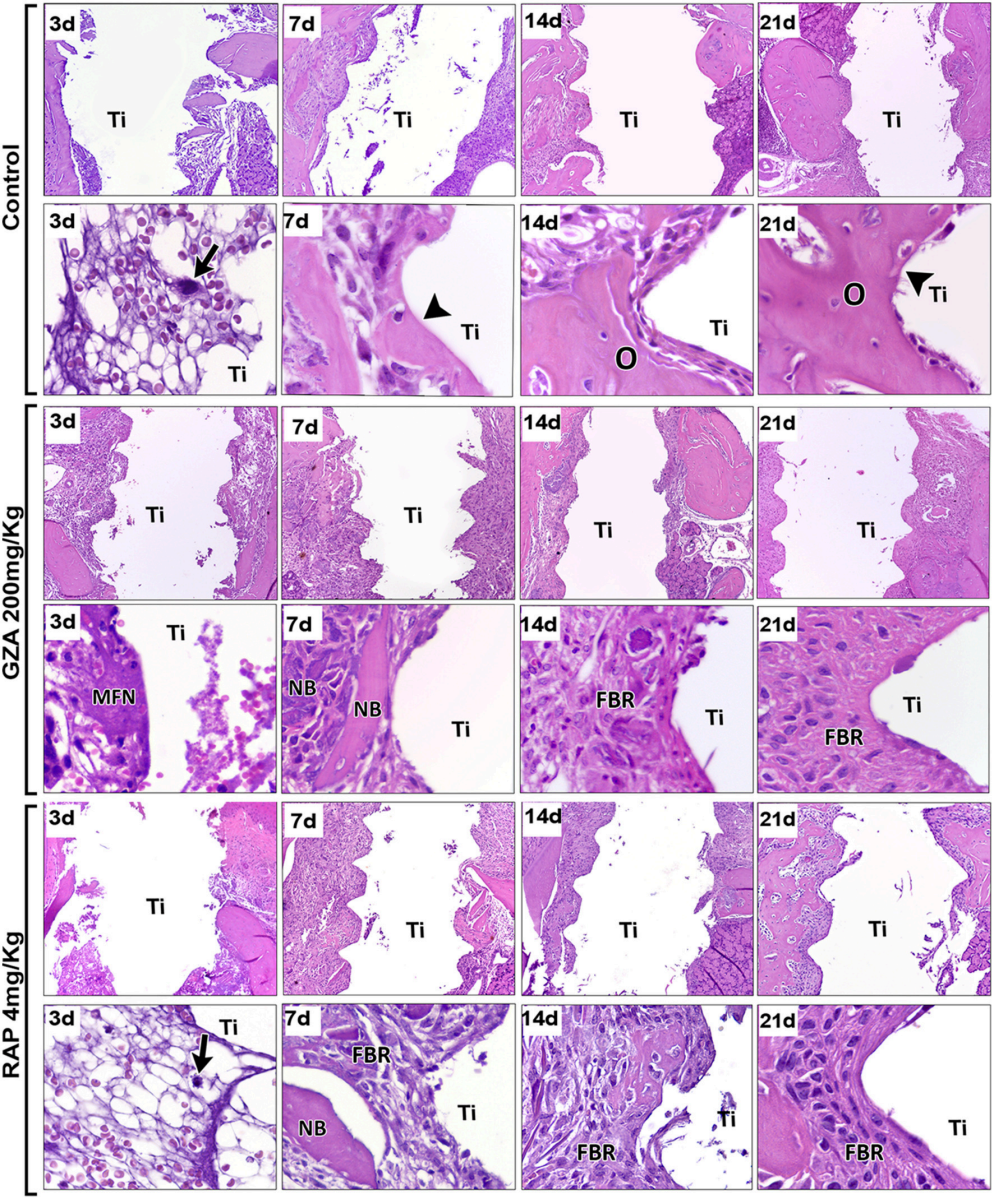
Histopathological analysis along oral osseointegration model in C57Bl/6 mice under HMGB1 or RAGE inhibition. Mice received Ti-screw implantation in the edentulous ridge of maxilla and were divided in according to each treatment: Control (C group, with no treatment); Glycyrrhizic Acid at a dosage of 200 mg/Kg/day (GZA group); or RAGE antagonistic peptide at dosage of 4 mg/Kg/day (RAP group). Chronology of oral osseointegration is observed throughout days 3, 7, 14, and 21 days. Histological slides were stained with H&E and images were captured at 10 and 100x magnification. Ti, Ti screw space; BC, Blood clot; Arrows, fibrin supporting cell migration; Arrowheads, bone/Ti contact region; O, osseointegration; MFN, Malformed fibrin network; NB, Necrotic bone; FBR, Foreign Body Reaction.

**Figure 5:**
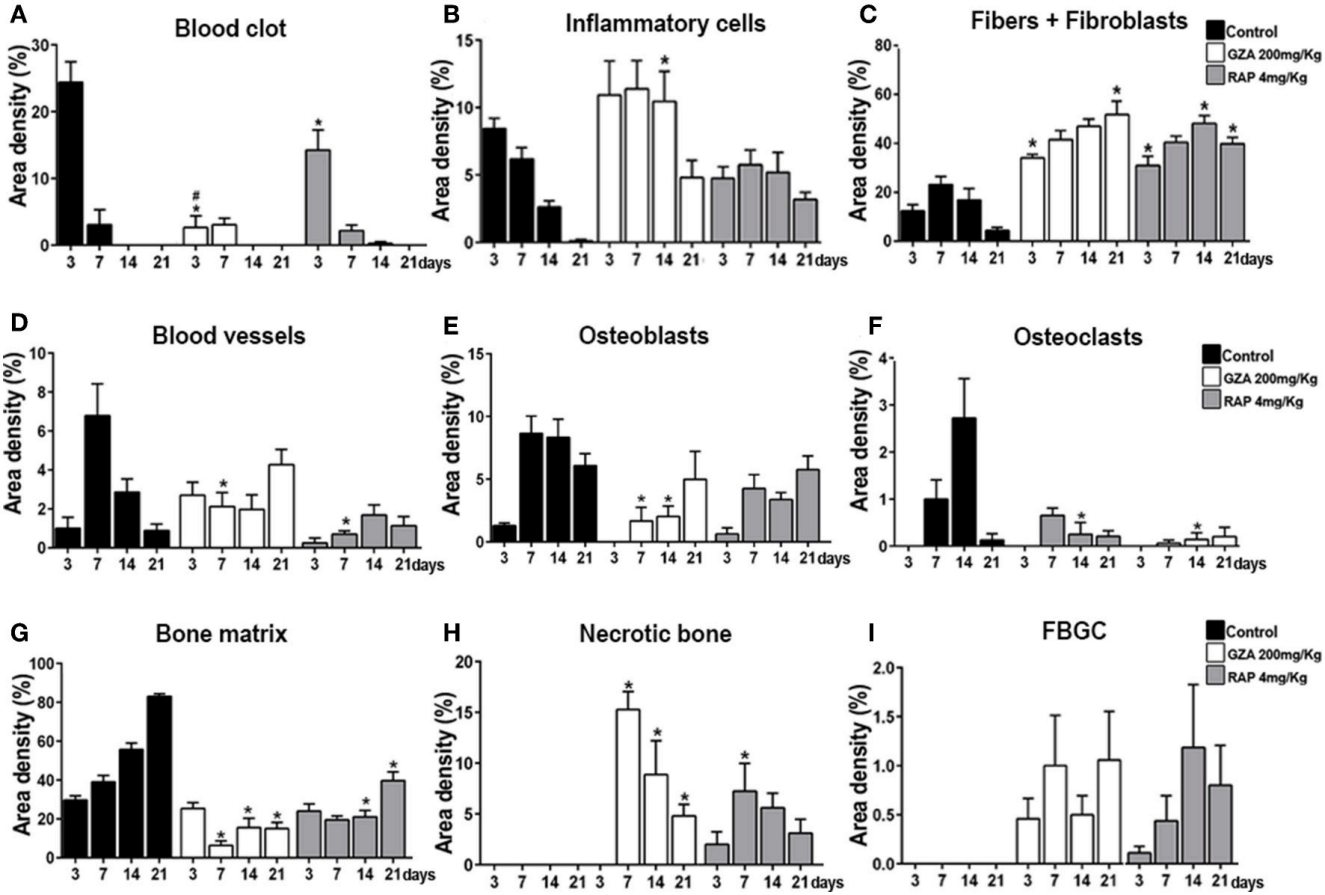
Histomorphometric analysis of healing components along oral osseointegration model in C57Bl/6 mice under HMGB1 or RAGE inhibition. Mice received Ti-screw implantation in the edentulous ridge of maxilla and were divided in according to each treatment: Control (C group, with no treatment); Glycyrrhizic Acid at a dosage of 200 mg/Kg/day (GZA group); or RAGE antagonistic peptide at dosage of 4 mg/Kg/day (RAP group). Results are presented as the means (± SD) of area density for each component related to osseointegration process: **(A)** Blood Clot, **(B)** Inflammatory cells, **(C)** Fibers + Fibroblasts, **(D)** Blood vessels, **(E)** Osteoblasts, **(F)** Osteoclasts, **(G)** Bone matrix, **(H)** Necrotic Bone, and **(I)** FBGC. Results are presented as the mean and SD from a total of six animals/samples (biological replicates) and nine semi-serial sections (technical replicates) *per* each group and time point. Symbol ^*^indicate a statistically significant difference vs. control, ^#^indicate differences between RAP and GZA groups (*p* < 0.05).

Histopathological analysis revealed osseointegration in the Control group, with intramembranous bone healing following overlapping phases from 3 to 21 days post Ti-implant placement in mice (Figure 4). Similar histological dynamics of osseointegration were observed in the GZA or RAP vehicle treated groups (data not shown). On the other hand, both experimental groups treated with RAP or GZA, exhibited failure of osseointegration, with the typical presence of fibrous connective tissue and foreign body giant cells (FBGC) formation at 14 and 21 days post-Ti implantation. At 3 days, the bone-implant interface in the Control group was filled predominantly by a blood clot (Figure 5A) providing support for cell infiltration (Figure 4, arrow). At 7 days, increased quantities of granulation tissue components were observed (blood vessels, fibroblasts, and fibers; Figures 5C,D), as well an initial differentiation of osteoblasts and bone matrix from the Ti threads and bone edges (Figure 4, arrowheads). At 14 and 21 days, granulation tissue components significantly decreased around Ti threads spaces, followed by an increased quantity of osteoblasts and bone matrix in the same regions (Figures 4, 5E,G) resulting in direct contact between implant and bone (Figure 4, arrowheads). Furthermore, Control and vehicle groups exhibited osteoclastic resorption lacunae and a few quantities of osteoclasts found around bone debris and pre-existing bone during 3 and 7 days post Ti implantation, followed by osteoclastic remodeling of newly formed bone at 14 and 21 days.

Comparatively to the osseointegration observed in the Control group, RAP treated mice also showed a suitable blood clot formation the bone-implant interface, but in a slighted reduced number, surrounded by an eosinophilic and slight matrix of fibrin network, with identifiable support for cell migration (Figure 4, arrows). On the other hand, the inhibition of HMGB1 in GZA treated mice resulted in a disorganized blood clot, with agglomerated platelets (#) and red blood cells separated from the malformed fibrin networks (MFN) (Figure 4, GZA group and Supplementary Figure 1) and a drastically reduced area density of this component (Figure 5A). Both RAP and GZA treated mice showed necrotic/non-viable bone persisting at 7–21 days post Ti-implantation, as well a foreign body reaction (FBR) with the presence of FBGC (Figures 4, 5H,I). The inhibition of RAGE in RAP group leaded to a negligible higher quantity of osteoblasts and bone formation in scattered areas surrounding Ti thread spaces compared to HMGB1 inhibition in GZA group (Figures 4, 5E). No statistical differences were observed in quantitative results for other elements (empty spaces, artifacts and Ti space; data not shown).

### Gene Expression Patterns in Osseointegration Under HGMB1 or RAGE Inhibition

**Figure 6:**
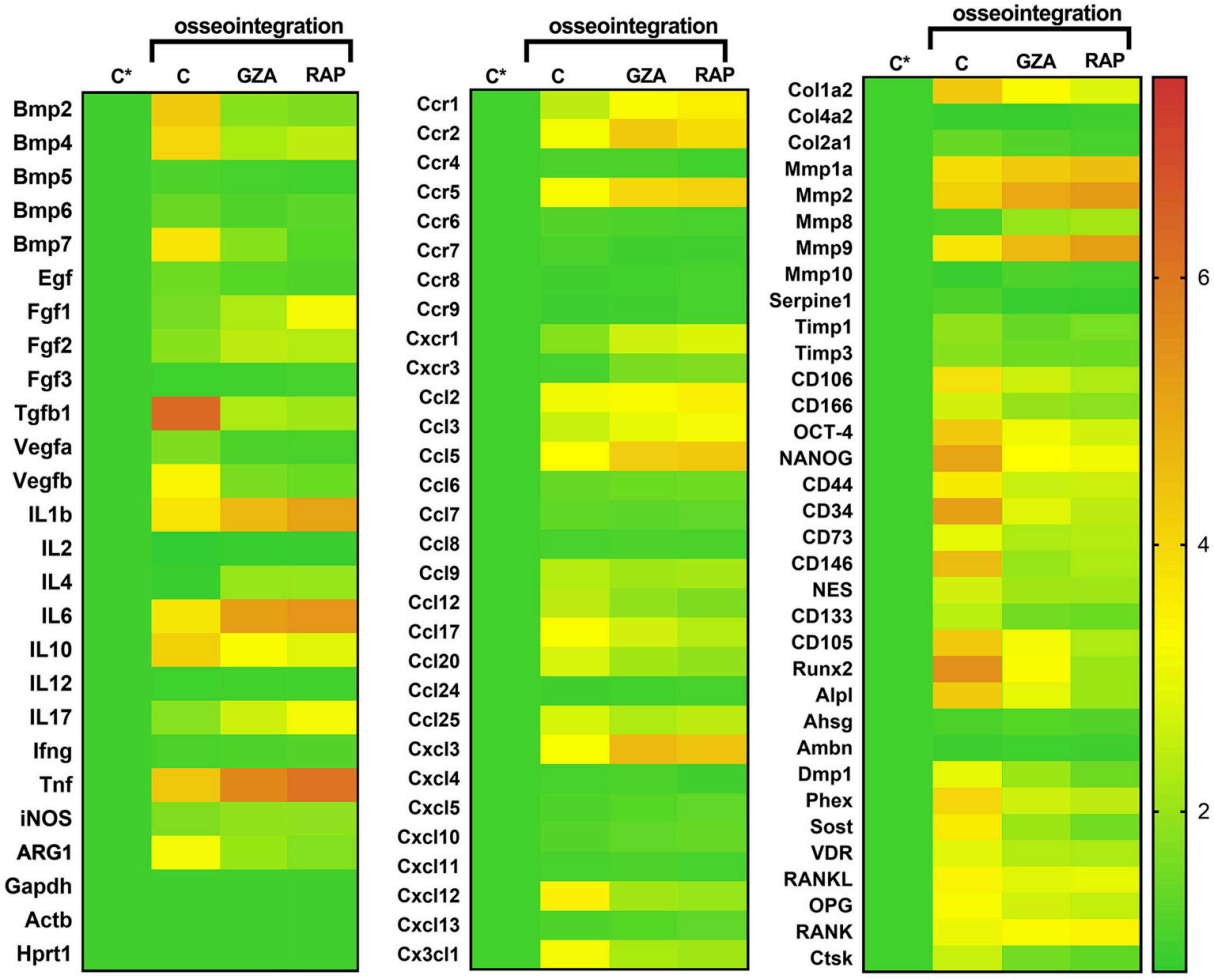
Gene expression patterns in the osseointegration sites under HMGB1 or RAGE inhibition. Mice received Ti-screw implantation in the edentulous ridge of maxilla and were divided in according to each treatment: Control (C group, with no treatment); Glycyrrhizic Acid at a dosage of 200 mg/Kg/day (GZA group); or RAGE antagonistic peptide at dosage of 4 mg/Kg/day (RAP group) Right side without Ti-screw implantation was used as tissue control and represented as C^*^. Molecular analysis of the gene expression patterns in the region of Ti screw implantation was comprised of an initial exploratory analysis by Real Time PCR array for each experimental group (Control, RAP and GZA), considering a pool of four samples (biological replicates) and two technical replicates from all the experimental periods (3, 7, 14, 21 days). Real Time PCR array analysis was performed with the VIA7 system (Applied Biosystems Limited, Warrington, Cheshire, UK) using a customized qPCR array comprised of the major targets from the Osteogenesis, Inflammatory Cytokines & Receptors and Wound Healing panels of the PCRarrayRT2 Profiler (SABiosciences/QIAGEN, Gaithersburg, MD, USA). Results are depicted as the fold increase change (and the standard deviation) in mRNA expression from triplicate measurements in relation to the control samples and normalized by internal housekeeping genes (GAPDH, HPRT, β-actin).

**Figure 7:**
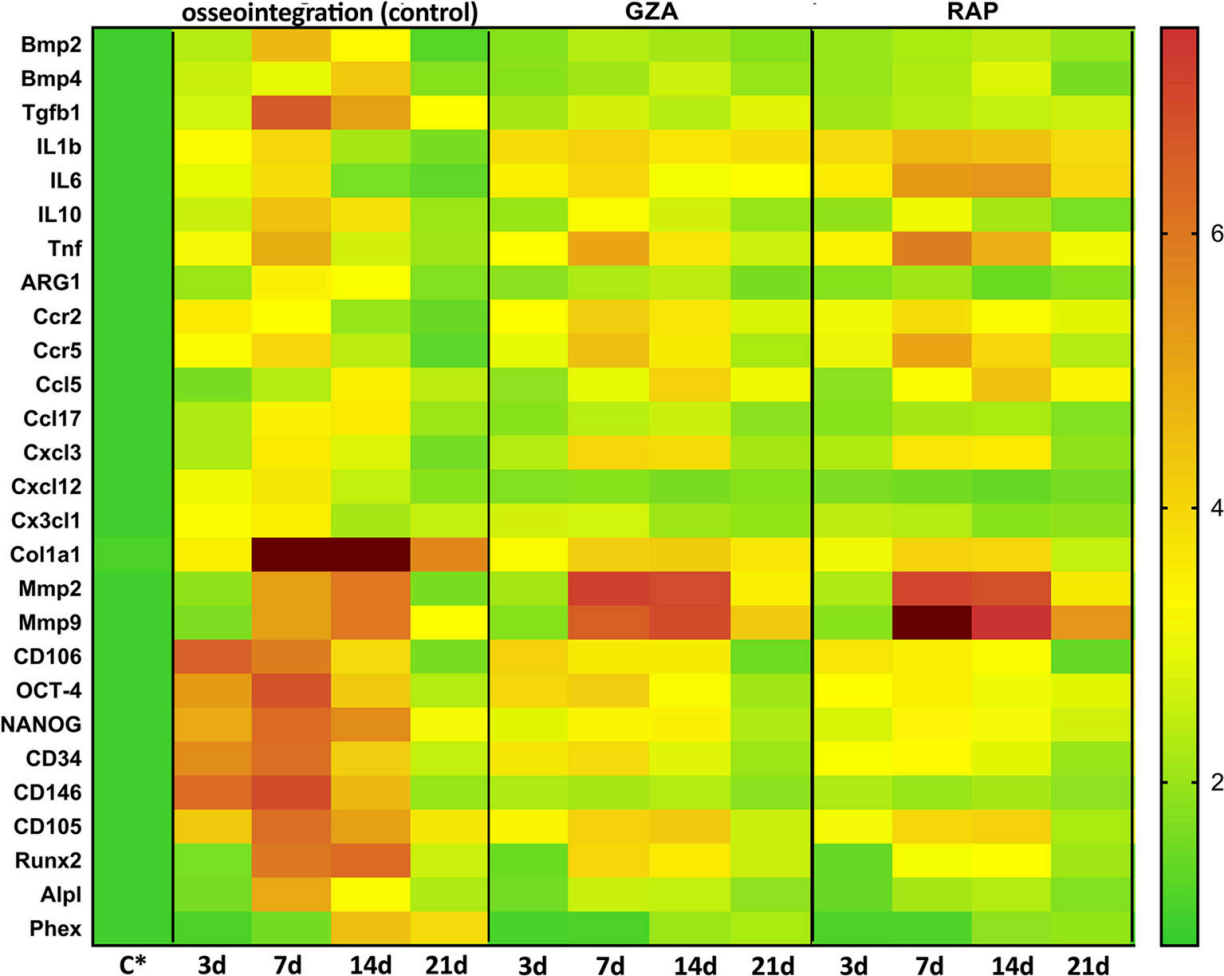
Kinetics of gene expression in the oral osseointegration sites under HMGB1 or RAGE inhibition. Mice received Ti-screw implantation in the edentulous ridge of maxilla and were divided in according to each treatment: Control (C group, with no treatment); Glycyrrhizic Acid at a dosage of 200 mg/Kg/day (GZA group) or RAGE antagonistic peptide at dosage of 4 mg/Kg/day (RAP group). Right side without Ti-screw implantation was used as tissue control and represented as C^*^. Molecular analysis of the gene expression in the region of Ti screw implantation was performed following each experimental time point (3, 7, 14, and 21 days), considering four samples (biological replicates) and two technical replicates *per* each group and time point. Targets with a significant expression variation in the previous Real Time PCR array from pooled samples were selected. Real Time PCR array analysis was performed with the VIA7 system (Applied Biosystems Limited, Warrington, Cheshire, UK) using a customized qPCR array comprised of the major targets from the Osteogenesis, Inflammatory Cytokines & Receptors and Wound Healing panels of the PCRarrayRT2 Profiler (SABiosciences/QIAGEN, Gaithersburg, MD, USA). Results are depicted as the fold increase change (and the standard deviation) in mRNA expression from triplicate measurements in relation to the control samples and normalized by internal housekeeping genes (GAPDH, HPRT, β-actin).

A pool of samples from all periods post-Ti implantation were initially analyzed by means of an exploratory Real Time PCR array (Figure 6), considering molecules involved in inflammatory response and bone healing (growth factors; immunological/inflammatory markers; extracellular matrix, MSC, and bone markers). Experimental groups (C, GZA, and RAP) were depicted as the fold increase change in relation to Control samples (C^*^), which are from the right side of maxilla of C57Bl/6 untreated mice, without surgery. Next, targets with a significant expression significant variation expression in pooled samples were analyzed according to their kinetics of expression during experimental periods (Figure 7).

For oral osseointegration model, among growth factors, TGFβ1, and VEGFb were significantly upregulated in C group, such as several MSC putative markers (OCT-4, NANOG, CD44, CD34, CD73, CD146, CD105, CXCL12); while the inhibition of HMGB1 (GZA group) and RAGE (RAP group) resulted in an important reduction in the mRNA levels for all these targets in pooled samples (Figure 6). Considering MSC putative markers, mRNA levels peaked at 3 and 7 days at osseointegration Control group and were significantly increased compared to GZA and RAP treated mice, as well TGFb and CXCL12. A slight upregulation for MSC markers were observed in GZA and RAP group compared to Control samples (C^*^).

Considering bone markers related to osteoblast differentiation (BMP2, BMP4, BMP7, Runx2, ALPL, DMP1, Phex, Sost, VDR) and bone remodeling (RANKL, OPG, CTSK), were positively upregulated in osseointegration Control group, whereas their expressions were drastically reduced in GZA and RAP group, as observed in pooled samples. On the other hand, RAP group presented an upregulation of FGF1 and FGF2 (Figure 6). In the osseointegration Control group, the kinetics of BMP2 mRNA levels peaked at 7 days and BMP4 peaked at 14 days. Runx2 and ALPL were upregulated at 7 and 14 days, significantly decreasing at 21 days, while Phex (a osteocyte differentiation marker) was upregulated at 14 days and 21 days (Figure 7).

Considering immunological markers for M1/M2 macrophages, a higher expression of ARG1 and IL10, markers for M2 phenotype, was particularly found in the osseointegration process of the Control group compared to the Control tissue (C^*^), but it was not observed in GZA and RAP treated mice (Figure 6). The mRNA levels of these M2 markers peaked at 7 and 14 days, as well TGFb in osseointegration Control group (Figure 7). The majority of chemokines and their receptors involved in inflammatory cells migration (CCR1, CCR2, CCR5, CCL2, CCL3, CCL5, CCL9, CCL12, CCL17, CCL20, CCL25, CXCL3, CXC3CL1) were upregulated in osseointegration sites in the Control group. On the other hand, GZA and RAP treated mice presented a higher expression of CCR2, CCR5, CCL5, and CXCL3 compared to the osseointegration C group in pooled samples (Figure 6). Also, pro-inflammatory cytokines were differentially expressed in osseointegration C group compared to the GZA and RAP groups (Figures 6, 7). While pro-inflammatory cytokines (IL1b, IL6, TNF), as well chemokine receptors (CCR2, CCR5) and chemokines (CCL5, CXCL3) were upregulated in early time points (3 and 7 days) in the osseointegration group, their mRNA levels remained upregulated in late time points (14 and 21 days) in GZA and RAP groups.

Finally, among the extracellular matrix markers, Col1a1, MMP2, and MMP9 were upregulated in all experimental groups (Figure 6). However, the kinetics of these markers were differently regulated comparing GZA an RAP groups to the osseointegration C group (Figure 7). In this way, mRNA levels of Col1a1 were significantly upregulated in the osseointegration Control sites at 7 and 14 days compared to GZA and RAP groups. On the other hand, GZA and RAP treated mice presented higher mRNA levels for MMP2 and MMP9 compared to the Control osseointegration sites (Figure 7).

### Histomorphometric, Birefringence, Immunohistochemical, and Molecular Analysis of Subcutaneous Healing Under Ti Implantation

**Figure 8:**
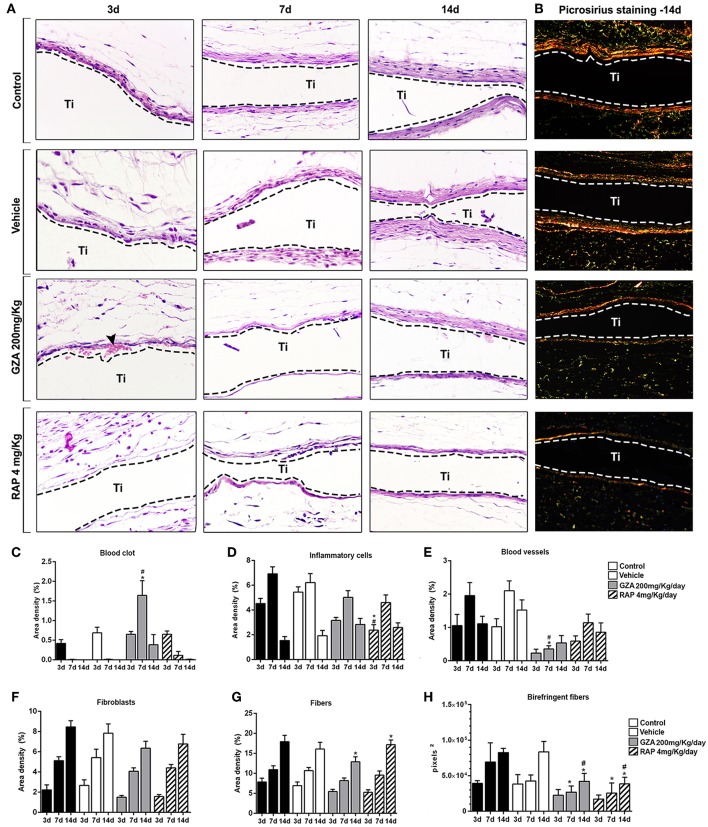
Histophatological, histomorphometric, and birefringence analysis of subcutaneous tissue post implantation of Ti-disc in C57Bl/6 mice under HMGB1 or RAGE inhibition. Mice received Ti-disc implantation in the subcutaneous tissue and were divided in according to each treatment: Control (C group, with no treatment); Vehicle (1.5% DMSO solution); Glycyrrhizic Acid at a dosage of 200 mg/Kg/day (GZA group); or RAGE antagonistic peptide at dosage of 4 mg/Kg/day (RAP group). Vehicle or drugs were administered 1 day before the surgical procedure and were given until the end of experimental periods (3, 7, and 14 days). **(A)** Comparative morphology of the healing phases post Ti disc implantation for each group, stained with H&E (40x magnification) and **(B)** Picrosirius red. **(C–G)** Results from histomorphometry of healing parameters (blood clot, inflammatory cells, fibroblasts, fibers, and blood vessels) are presented as the mean of area density for each structure measured in each examined group. Results are presented as the mean and SD from a total of five animals/samples (biological replicates) and eight semi-serial sections (technical replicates) *per* each group and time point. **(H)** Intensity of birefringence performed using image-analysis software (AxioVision, v. 4.8, CarlZeiss) for total area of birefringent collagen fibers (pixels^2^). Results are presented as the mean and SD from a total of five animals/samples (biological replicates) and four semi-serial sections (technical replicates) *per* each group and time point. **(C–H)** Symbols indicate statistically significant difference (*p* < 0.05) between experimental groups (GZA and RAP) vs. Control^*^ and experimental groups vs. Vehicle^#^ at the same time point.

**Figure 9:**
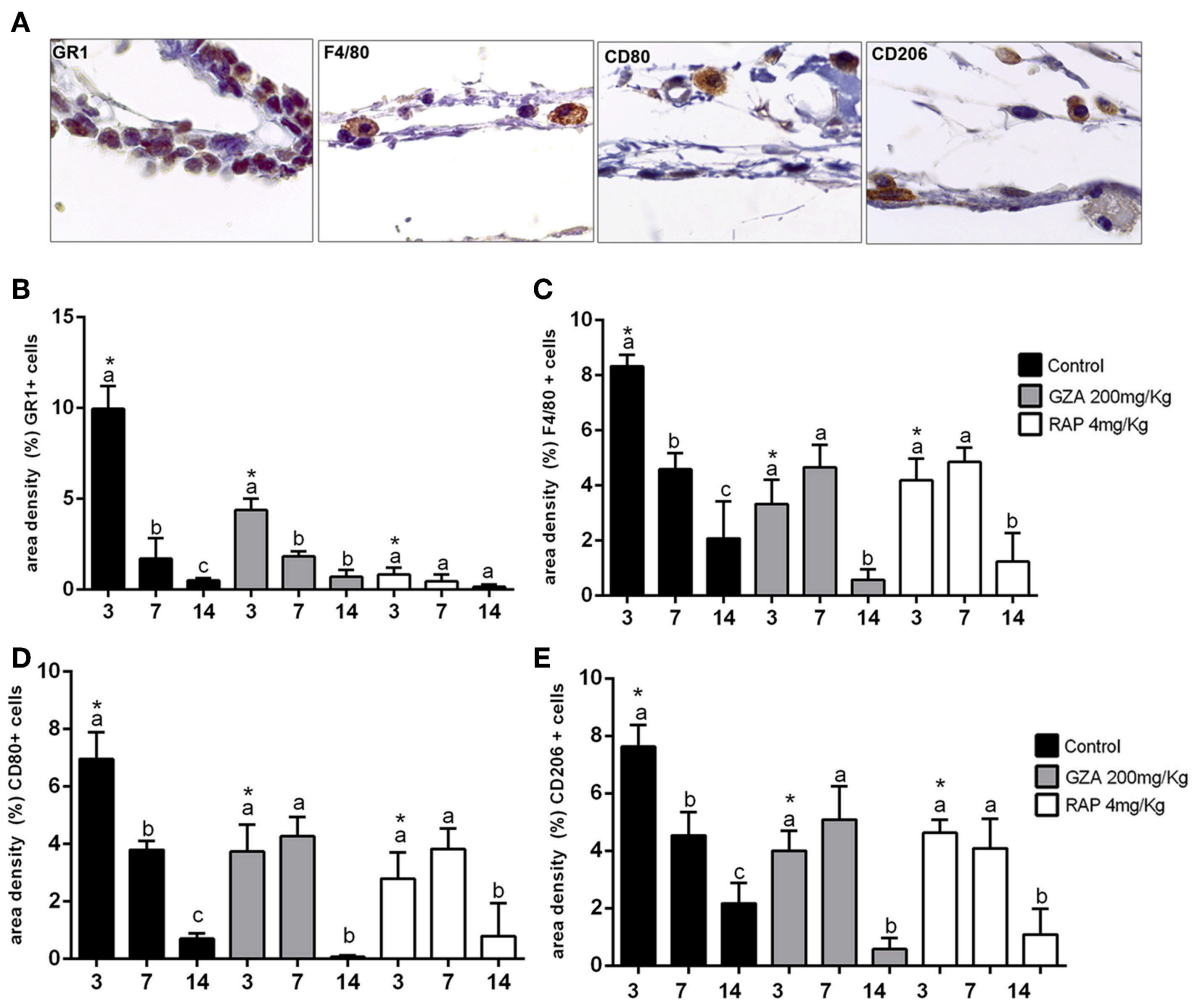
Inflammatory cells recruited to the Ti disc implantation sites in C57Bl/6 mice treated with HMGB1 inhibitor or RAGE antagonist. Mice received Ti-disc implantation in the subcutaneous tissue and were divided in according to each treatment: Control (C group, with no treatment); Vehicle (1.5% DMSO solution); Glycyrrhizic Acid at a dosage of 200 mg/Kg/day (GZA group); or RAGE antagonistic peptide at dosage of 4 mg/Kg/day (RAP group). Vehicles or drugs were administered 1 day before the surgical procedure and were given until the end of experimental periods (3, 7, and 14 days). **(A)** Representative sections of 3 days time point post Ti implantation. Quantitative analysis of **(B)** GR1+, **(C)** F4/80+, **(D)** CD80+cells and **(E)** CD206+ cells was performed for each group at days 3, 7, and 14 days post Ti implantation. Results are presented as the mean and SD from a total of five animals/samples (biological replicates) and three semi-serial sections (technical replicates) *per* each group and time point. Different letters indicate significant differences in each time point (*p* < 0.05); symbol ^*^indicate significant differences between experimental groups (GZA and RAP) vs. control at the same time point.

Control and both GZA and RAP control vehicle treated mice showed a suitable blood clot formation and a slight inflammatory infiltrate at 3 days, followed by a dense connective tissue formation, containing fibroblasts and negligible quantities of inflammatory cells surrounding region of Ti-disc implantation at 14 days (Figure 8A). Also, birefringence analysis revealed a yellow/red spectrum of collagen fibers surrounding the Ti at 14 days (Figure 8B). On the other hand, the inhibition of HMGB1 by GZA treatment caused a disruption of blood clot formation at 3 days (arrow, Figure 8) and a persistence of blood clot and a decreased area density of blood vessels around Ti disc implantation at 7 days (Figures 8C,E). Similarly, both treatments (the inhibition of HMGB1 and the antagonism of RAGE), impaired the host response to the Ti disc by a decreased collagen fiber formation compared to the control and control vehicles, but with no negative effects in the amount of fibroblasts (Figures 8F,G). The reduced tissue repair in GZA and RAP could be mainly associated with an ineffective inflammatory response caused by the inhibition of inflammatory signals induced by HMGB1 and RAGE. Immuhistochemistry of GZA and RAP group showed a drastic reduction of GR1+ cells and macrophages (F4/80+ cells, CD80+ cells, CD206+ cells) migration toward the implantation sites at 3 days post Ti implantation compared to the Ti control group (Figures 9A–E).

In parallel and in agreement with molecular results for oral osseointegration model, the gene expression patterns in subcutaneous implanted sites on Ti control was also revealed growth factors involved in cell proliferation (FGF1, FGF2, FGF3, TGFb1, EGF) and angiogenesis (VEGFa,b) significantly up-regulated in the Ti Control group compared to the endogenous control (Supplementary Figure 2). Consistently, tissue healing and maturation of the ECM was also evidenced in Ti control by a high upregulation of ECM remodeling markers, such as the matrix metalloproteinases (MMP1a, MMP2, MMP9) and their tissue inhibitors TIMPs (TIMP1, TIMP3), as well the protease cathepsin G (CTSG). Among the upregulated cytokines in Ti control samples, CXCL10, CXCL12, CXCL11, IL1β, IL6, TNF were up regulated in the inflammatory phase of healing. Growth factors involved in cell proliferation, mainly for FGF family, were up regulated in GZA and RAP, such as in the Control group, while several ECM formation (Col1a2, Col2a1) and remodeling markers (MMP1a, MMP2, MMP9, TIMP1, TIMP3, CTSG) were down regulated GZA and RAP compared to the control. Importantly, GZA and RAP group presented a downregulation of molecules involved in cell adhesion and migration (CTGF, VTN, ITGA2, ITGA4, ITGA5). All together, these results indicate a role of HMGB1 and RAGE on fibroblasts migration, differentiation, and matrix deposition along tissue repair surrounding a classic biomaterial.

## Discussion

Among the several DAMPs and their accompanying PRRs, the interaction of HMGB1 with the receptor RAGE has been associated with the activation of inflammatory responses and wound healing, especially in non-infectious environments (18, 19, 22). In this study, the possible involvement of the HMGB1/RAGE pathway in the modulation of host inflammatory immune response at Ti/host interface, and the subsequent influence in the healing and osseointegration processes were investigated. Therefore, to determine the role of HMGB1 and RAGE in the osseointegration process in a cause-and-effect manner, C57Bl/6 mice were subjected to Ti-implant surgical placement in the maxillary edentulous area and were treated with GZA and RAP, respectively, an HMGB1 inhibitor (41) and a RAGE inhibitor (42).

Initially, our results demonstrated that HMGB1 was present in the protein adsorption layer characteristically formed in biomaterials surface after implantation, in both Ti-screws implanted in bone and Ti-discs implanted in subcutaneous tissue (Figure 1). Importantly, despite the general assumption that endogenous DAMPs are released upon biomaterials implantation, this is the first actual demonstration that DAMPs (specifically HMGB1) are in fact released and can adsorb to Ti surface. The kinetics of HMGB1 release and adsorption is in agreement with the hypothesis of the injury-triggered release, characterized by high levels in the initial time point followed by a gradual decrease over time (50–52). Also, our results demonstrated that inhibition of both, HMGB1 and RAGE, impaired Ti-mediated osseointegration, as demonstrated by the critical alterations in the dynamics of mineralized and organic bone matrix formation (Figures 2, 3). Accordingly, the inhibition of HMGB1 in a model of tooth extraction in mice significantly delayed the bone healing process, but without inhibiting it completely (21). However, it is crucial to consider that in the present study, the presence of a biomaterial is an important variable in the healing site; which may account for the complete impairment of the osseointegration process in comparison with the partial influence of HMGB1 inhibition described in the socket healing (21). Importantly, no previous studies have described possible associations between RAGE blockade and bone healing or osseointegration. It is also important to mention that HMGB1 and RAGE blockade impair the healing of subcutaneous tissue after the grafting of a Ti-disc, reinforcing the role of HMGB1/RAGE axis in the host response to biomaterials and in the subsequent healing response. While the subcutaneous implantation of Ti-devices obviously does not mimic the osseointegration response, it have been considered a valuable model to study biomaterial/host interaction (53), which can be very useful, especially in mice in the view of the very limited dimensions of the Ti implant used for osseointegration analysis, which limits some experimental approaches.

In order to investigate the mechanisms underlying impaired osseointegration due HMGB1 and RAGE inhibition, a series of histomorphometric, and molecular analysis were performed, comparing unsuccessful and successful osseointegration sites. The process of osseointegration starts with the surgical preparation of the bone niche/defect for implant placement, when coagulation proteins from blood are released and then activated to provide the clot formation and consequently a provisional matrix for cell recruitment and migration (15). Accordingly, in the Control group (which achieved osseointegration), an organized blood clot was evidenced at the host/Ti interface at 3 days post-implantation. However, HMGB1 inhibition resulted in disruption of fibrin network formation and impairment of the blood clot structure, followed by a significant decrease in blood clot area density when compared to the Control group. Indeed, HMGB1 acts synergistically with thrombin to promote fibrin deposition and accelerate the coagulation *in vivo*, evidencing its role as an organizer in post-injury wound healing (54). Thus, the initial event of osseointegration impairment due to GZA administration seems to be primarily related to the disruption of the blood clot, since the establishment of a fibrin network in association with Ti threads spaces was drastically compromised upon HMGB1 inhibition. Additionally, RAGE inhibition also resulted in a reduction of blood clot area density when compared to the Control group, but without drastic effects over clot organization as observed upon HMGB1 inhibition. Accordingly, while HMBG1 seems to also act in the clotting process directly (i.e., in a RAGE independent way), RAGE expressed on platelets surface is associated with their activation by DAMPs (HMGB1 and S100 proteins) and platelet aggregation (55, 56), which consequently influence the clotting process, but also the release of additional HMGB1 and other pro inflammatory molecules (56, 57).

In addition to the initial interferences in the clotting process, previous studies demonstrated that HMGB1 promotes the secretion of multiple cytokines in the injured sites, strongly activating and driving the acute inflammatory response (58). Also, it is important to consider that HMGB1 is supposed to play also biphasic actions on injured sites (pro-inflammatory activity or immune tolerance/healing) depending of the environment redox state of its three conserved cysteines (Cys23, Cys45 [Box A], and Cys106 [BoxB]) (23). In this context, it has been proposed that during acute inflammatory response, the release of ROS/RNS induce the active and proinflammatory form of HMGB1 (reduced form of HMGB1); while the oxidation of HMGB1 cause immune tolerance, allowing the healing (22, 23). Considering the receptor RAGE, it is important to mention that two extracellular secreted forms of RAGE can be also present in the environment, besides the conventional receptor, they are endogenous secretory (es) and soluble (s) RAGE, have been identified and play active roles on skeletal biology, mainly related to osteoporosis in aged mice (59). It has been supposed that these RAGE isoforms (mainly sRAGE), could also their ligand-binding ability, acting as decoy receptors preventing ligand binding to RAGE. Importantly, while the analysis of redox modulation of HMGB1 activities, as well of a putative role for sRAGE, are beyond the scope of the present study, since our data point to a role for HMGB1 in osseointegration process, future specific studies focused in such elements may provide additional interesting information to the field.

In this study, HMGB1 or RAGE inhibition disturbed the natural course/fate of inflammatory response after Ti implantation. This resulted in the persistence of inflammatory cells around Ti threads until latter time points, comprising primarily macrophages as suggested by the cellular morphology, while Control mice exhibited the resolution of a transient inflammatory response in early time points (Figure 4). Accordingly, the molecular analysis demonstrated that HMGB1 or RAGE inhibition resulted in a persistence of high mRNA levels of CCR2, CCR5, CCL5, which are mainly associated with macrophage migration (48, 60), as well as pro-inflammatory cytokines (IL1b, IL-6, and TNF) that characterize M1 activity (61). Thus, our findings suggest a role of HMGB1 and RAGE on the modulation/resolution of chronic inflammatory response post Ti implantation, probably affecting the overall M1/M2 macrophages response. While the reduced size of the Ti-device limits some additional analysis, the subcutaneous implantation of Ti-discs allowed the characterization of the inflammatory changes upon HMGB1 and RAGE blockade, and demonstrate that the total macrophages, M1 and M2 cells counts were reduced in the absence of a functional HMGB1/RAGE axis. Macrophages are considered key elements in the connection between inflammatory and healing events (11). The initial presence of M1 macrophages has been implicated as an essential step for the activation of acute inflammatory response, while the transitory presence of M2 cells in the proliferative/regenerative phase has been suggested as favorable for the regenerative outcome (11). Conversely, a prolonged M1 activity has been associated with negative outcomes of biomaterial implantation, such as chronically inflamed tissue and severe foreign body reaction (FBR) (37). Considering the osseointegration and subcutaneous results, it is possible to suggest that HMGB1/RAGE axis is required for a proper macrophage chemoattraction after Ti implantation, and that both M1 and M2 responses, and the natural M1/M2 switch along the healing, are compromised by HMGB1 and RAGE inhibition. Accordingly, the molecular analysis of the successful Ti-osseointegration sites in the Control group demonstrated an initial M1-type response followed by a M2-type switch, evidenced by upregulation of M2-type markers (ARG1, TGFb, IL10, and CCL17) (62), which were disrupted by HMGB1/RAGE blockade. These observations are in agreement with previous studies (4, 5, 63). In view of that, the provision of environmental cues that govern the phenotype switch of macrophages and different healing outcomes post biomaterial implantation have been usually based on the biomaterial properties, in a perspective where Ti-based devices might modulate or allow a favorable M1/M2 switch (9). However, we demonstrated that inhibition of a DAMP or its receptor (HMGB1 or RAGE) following biomaterial implantation, can also drastically affect the initial microenvironmental signals for triggering osseointegration, even using a gold standard biomaterial such as a Ti-based device. Significantly, the effects of HMGB1 or RAGE inhibition are not limited to macrophages, as demonstrated by the significant reduction of granulocytes (Gr1+ cells) in the Ti disc implantation. While the main of focus in the cellular aspects of osseointegration (and in the biomaterials in general) have been over macrophages (9, 62), granulocytes are essential elements of early host response (64), and consequently can also theoretically impact the subsequent healing and osseointegration.

The lack of favorable biological microenvironment signals in biomaterial implantation sites can result in persistent chronic inflammation, consequently driving the wound healing around the biomaterial into a foreign body response (FBR) (65). In this manner, HMGB1 and RAGE inhibition drastically reduced the expression of MSC markers and bone markers in the sites of Ti implantation, which was reflected in a fibrotic outcome surrounding Ti threads (Figures 6, 7), with features of FBR, such as differentiation of FBGC surrounding the biomaterial and non-viable bone (Figure 4), increased expression of MMPs (Figure 7), followed by fibrous tissue formation and consequent biomaterial encapsulation (Figure 4). As previously proposed by literature, the modulation of host response for desirable biomaterial incorporation outcome is in part surface-based, depending on beneficial biomaterial properties, but signals provided from biomaterial implantation trauma have also been suggested as crucial cues in this process (9). Accordingly to the Control group results, in the presence of a constructive set of external and endogenous factors, including Ti as the external factor and HMGB1 and RAGE as part of endogenous factors, the inflammatory signals triggered post Ti implantation was linked to upregulation of MSC markers (CD206, OCT-4, NANOG, CD44, CD34, CD73, CD146, CD105) at earlier time points (3 and 7 days), and subsequent bone cells differentiation (Runx2, Alp), bone matrix deposition (Col1a1), remodeling (MMP2 and MMP9) (45), and bone maturation (Phex) (66) (Figure 7).

**Figure 10:**
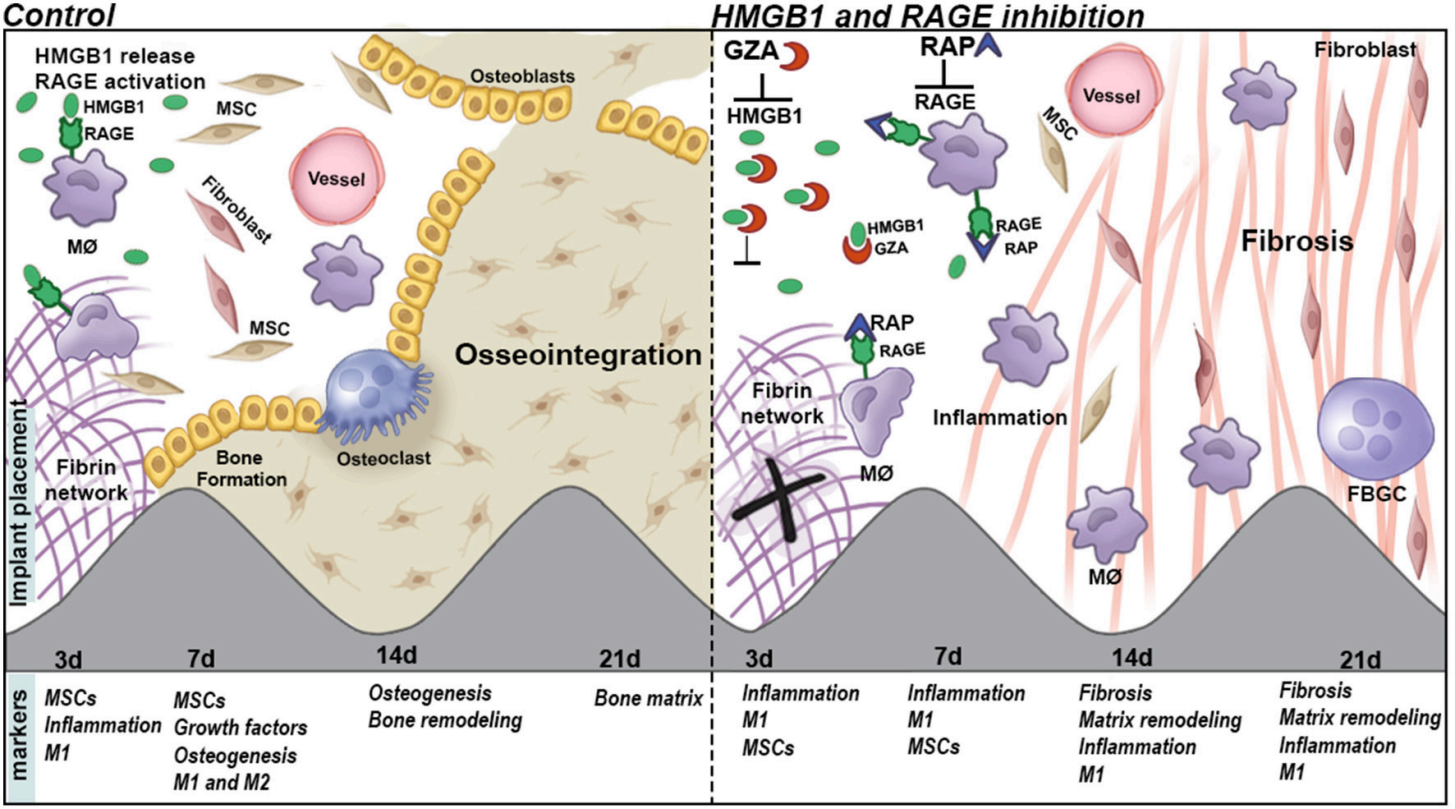
Graphical abstract of proposed roles of HGMB1 and RAGE along oral Ti osseointegration process in mice.

The body of this work suggests the participation of HMGB1 in multiple stages of osseointegration process, as a blood clot organizer and inflammatory/healing molecule (Figure 10). Several studies have suggested that HMGB1 can act as a regenerative mediator, by triggering inflammation (20), but also as a healing organizer, promoting the recruitment of MSCs, and platelets activation (20, 58). In this cause-effect study, the inhibition of extracellular HMGB1 following biomaterial implantation caused failure of Ti-mediated osseointegration (Figures 2–5), which could be associated to its multiple roles acting as a biochemical mediator for clot formation (54, 56, 67), as well as by triggering of signaling inflammatory pathways, which involve the activation of different receptors, such as RAGE. In fact, under the inhibition of RAGE, the immediate extracellular effects of released HMGB1 were maintained, such as confirmed by a suitable blood clot structure in the osseointegration sites at 3 days compared to the HMGB1 inhibition group. However, under the inhibition of RAGE, the HMGB1 cellular effects related to HMGB1/RAGE pathway was blockade, which also resulted in unsuccessful osseointegration.

It is also important to consider the despite the fact that a clear biological effect was observed upon the administration of the RAP and GZA in this study, the dosages used for both inhibitors were based in previous studies carried in C57Bl/6 mice but in different models and kinetics of drug administration (41, 42). Despite the effects observed in our study are compatible with the biological role of HMGB1 and RAGE, confirming the effectiveness of both inhibitions and demonstrating a role for HMGB1 and RAGE in osseointegration process, future studies including a dose-response analysis, may provide additional interesting information to the field. Finally, future studies are required to investigate the inhibition of HMGB1 and/or RAGE only in initial time points during Ti-mediated osseointegration, when these molecules are prevalent and theoretically mainly required, in order to determine their role in each phase of osseointegration.

## Conclusion

Taken together, our findings suggest that HMGB1 and RAGE actively influence the osseointegration process, by their influence in the balance of host inflammatory immune response, which includes macrophages migration and M1/M2 response, MSC markers expression, and bone deposition (Figure 10).

## Author Contributions

CB and GG contributed to the conception and design, the acquisition, analysis, and interpretation, drafted the manuscript, critically revised the manuscript, gave final approval, and agreed to be accountable for all aspects of work. FC contributed to the acquisition, analysis, and interpretation, drafted the manuscript, critically revised the manuscript, gave final approval, and agreed to be accountable for all aspects of work. ES, APT, and CF contributed to the acquisition, analysis, and interpretation, drafted the manuscript, gave final approval, and agreed to be accountable for all aspects of work. RT, AC, APFT, and DR contributed to the acquisition, analysis, and interpretation, gave final approval, and agreed to be accountable for all aspects of work.

### Conflict of Interest Statement

The authors declare that the research was conducted in the absence of any commercial or financial relationships that could be construed as a potential conflict of interest.

## Supplementary Material

Supplementary Figure 1 Histopathological analysis of blood clot in C57Bl/6 mice at 3 days post Ti implantation. Mice received Ti-screw implantation in the edentulous ridge of maxilla and were divided in according to each treatment: Control (C group, with no treatment); Glycyrrhizic Acid at a dosage of 200 mg/Kg/day (GZA group); or RAGE antagonistic peptide at dosage of 4 mg/Kg/day (RAP group). Blood clot is observed throughout days 3, 7, 14, and 21 days. Histological slides were stained with H&E and images were captured at 10 and 100x magnification.

Supplementary Figure 2 Gene expression patterns post subcutaneous Ti disc implantation in C57Bl/6 mice treated with HMGB1 inhibitor or RAGE antagonist. Mice received Ti-disc implantation in the subcutaneous tissue and were divided in according to each treatment: Control (C group, with no treatment); Glycyrrhizic Acid at a dosage of 200 mg/Kg/day (GZA group); or RAGE antagonistic peptide at dosage of 4 mg/Kg/day (RAP group). Four biological replicates from subcutaneous tissue samples were removed at 3, 7, and 14 days post Ti implantation and a pool of samples from all the experimental time periods in each experimental group was used for a gene expression pattern analysis. Samples of subcutaneous tissue without surgery were used as control. Two technical replicates were considered for each assay. Gene expression was performed by using exploratory analysis by Real Time PCR array, with the VIA7 system (Applied Biosystems, Warrington, UK) and a customized qPCR array comprised of the major targets (Inflammatory Cytokines & Receptors and Wound Healing panels) of the PCRarrayRT2 Profiler (SABiosciences/QIAGEN). Results are depicted as the fold increase change (and the standard deviation) in mRNA expression from triplicate measurements in relation to the control samples and normalized by internal housekeeping genes (GAPDH, HPRT, β-actin).
