# Supplementary figures and images for: HGMB1 and RAGE as Essential Components of Ti Osseointegration Process in Mice
Source: Front Immunol. 2019 Apr 5;10:709. doi: 10.3389/fimmu.2019.00709 (PMC6461067; doi:10.3389/fimmu.2019.00709)

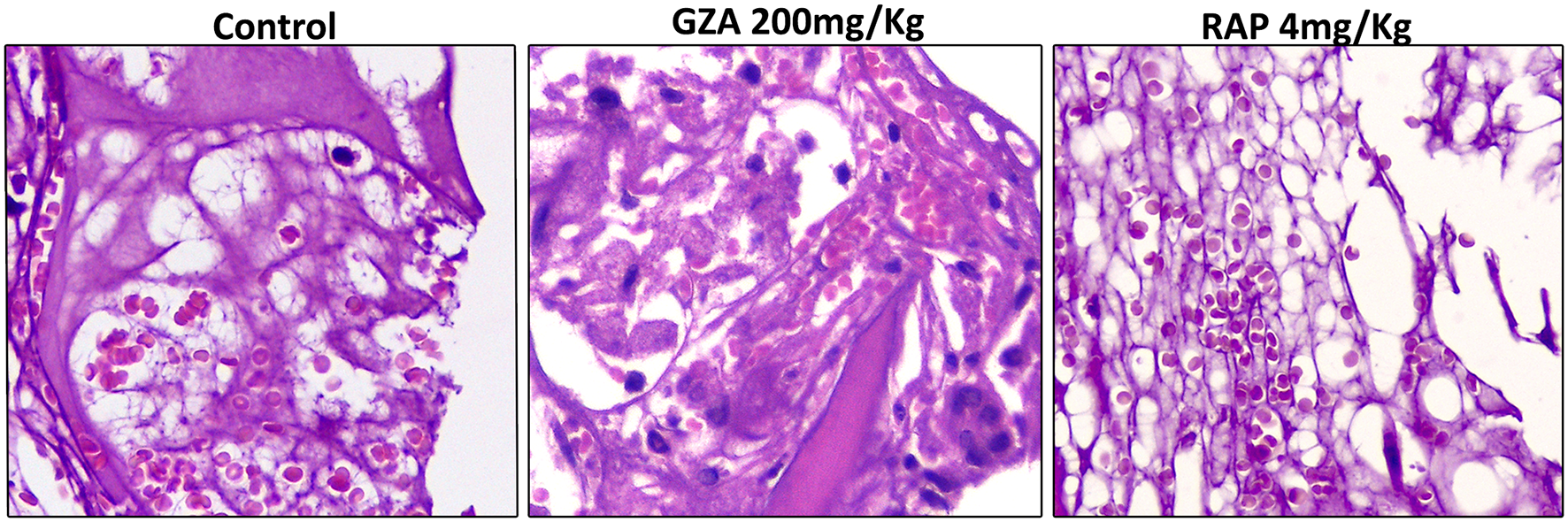

Supplement: Supplementary Figure 1 — Histopathological analysis of blood clot in C57Bl/6 mice at 3 days post Ti implantation. Mice received Ti-screw implantation in the edentulous ridge of maxilla and were divided in according to each treatment: Control (C group, with no treatment); Glycyrrhizic Acid at a dosage of 200 mg/Kg/day (GZA group); or RAGE antagonistic peptide at dosage of 4 mg/Kg/day (RAP group). Blood clot is observed throughout days 3, 7, 14, and 21 days. Histological slides were stained with H&E and images were captured at 10 and 100x magnification. [file Image_1.TIF]

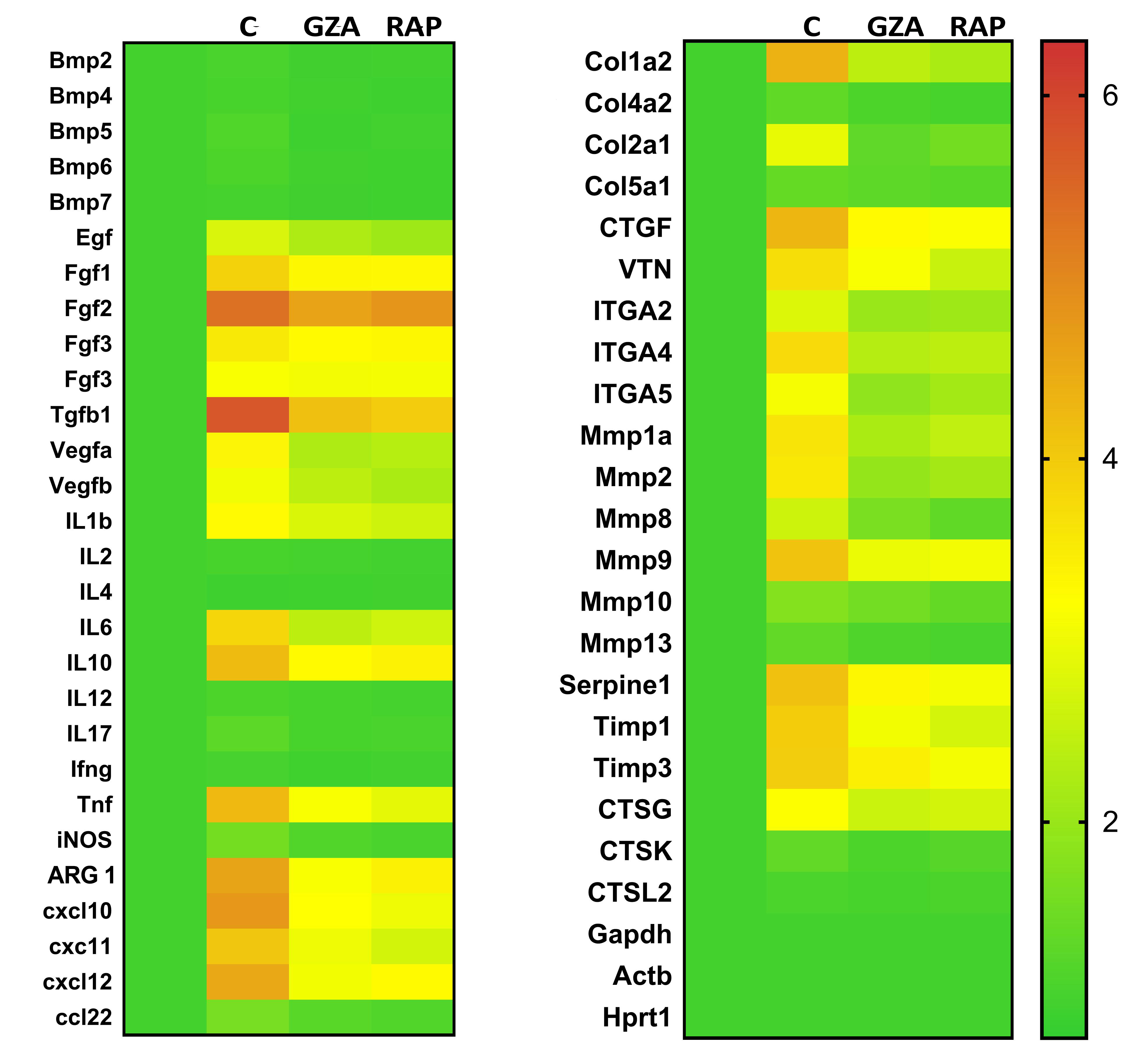

Supplement: Supplementary Figure 2 — Gene expression patterns post subcutaneous Ti disc implantation in C57Bl/6 mice treated with HMGB1 inhibitor or RAGE antagonist. Mice received Ti-disc implantation in the subcutaneous tissue and were divided in according to each treatment: Control (C group, with no treatment); Glycyrrhizic Acid at a dosage of 200 mg/Kg/day (GZA group); or RAGE antagonistic peptide at dosage of 4 mg/Kg/day (RAP group). Four biological replicates from subcutaneous tissue samples were removed at 3, 7, and 14 days post Ti implantation and a pool of samples from all the experimental time periods in each experimental group was used for a gene expression pattern analysis. Samples of subcutaneous tissue without surgery were used as control. Two technical replicates were considered for each assay. Gene expression was performed by using exploratory analysis by Real Time PCR array, with the VIA7 system (Applied Biosystems, Warrington, UK) and a customized qPCR array comprised of the major targets (Inflammatory Cytokines & Receptors and Wound Healing panels) of the PCRarrayRT2 Profiler (SABiosciences/QIAGEN). Results are depicted as the fold increase change (and the standard deviation) in mRNA expression from triplicate measurements in relation to the control samples and normalized by internal housekeeping genes (GAPDH, HPRT, β-actin). [file Image_2.JPEG]
